# Timing of pharmacological thromboprophylaxis in severe traumatic brain injury: a systematic review and meta-analysis

**DOI:** 10.1007/s00701-026-07002-2

**Published:** 2026-08-14

**Authors:** Midhun Mohan, Santosh Guru, Siddarth Kannan, Zak Farazi, Abdel-Rahman Abdel Fattah, Conor Gillespie, Edoardo Viaroli, Muhammad Shuaib Khan, Charikleia S. Vrettou, Stefanos Korfias, Daniel Horner, Virginia Newcombe, Peter Hutchinson, Angelos Kolias

**Affiliations:** 1 Division of Neurosurgery, Department of Clinical Neurosciences, University of Cambridge, Cambridge, UK; 2 The Walton Centre, Liverpool, UK; 3 University of Lancashire, Preston, UK; 4 School of Clinical Medicine, University of Cambridge, Cambridge, UK; 5 First Department of Critical Care Medicine, National & Kapodistrian University of Athens, Evangelismos Hospital, Athens, Greece; 6 First Department of Neurosurgery, National & Kapodistrian University of Athens, Evangelismos Hospital, Athens, Greece; 7 Critical Care Department, Northern Care Alliance NHS Foundation Trust, Salford, UK; 8 Division of Immunology, Immunity to Infection and Respiratory Medicine, University of Manchester, Manchester, UK; 9 Perioperative, Acute, Critical, and Emergency Care (PACE) Section, Department of Medicine, University of Cambridge, Cambridge, UK

**Keywords:** Traumatic brain injury, Venous thromboembolism, Thromboprophylaxis, Deep vein thrombosis, Intracranial haemorrhage, Meta-analysis

## Abstract

**Purpose:**

Traumatic brain injury (TBI) presents competing risks of venous thromboembolism (VTE) and intracranial haemorrhage progression, rendering pharmacological thromboprophylaxis (PTP) timing uniquely contentious. The dilemma is most acute in severe TBI, where both risks are greatest. This systematic review and meta-analysis (PROSPERO: CRD420261388300) evaluates early versus late PTP on VTE, intracranial haemorrhage progression, and mortality in adults with severe TBI, applying a strict severity definition (Glasgow Coma Scale (GCS) 8 or less in 100% of the analysed cohort) to confirm and refine findings from prior mixed-severity syntheses.

**Methods:**

MEDLINE, EMBASE, and CENTRAL were searched from inception to 1 May 2026. Studies enrolling adults with severe TBI (GCS 8 or less throughout analysed cohort) were eligible. Primary outcomes were VTE, intracranial haemorrhage progression, and in-hospital mortality. Definitions of VTE, and timing thresholds for early and late PTP were those used by the investigators in the individual studies. Risk of bias and certainty of evidence were assessed using ROBINS-I and GRADE. Random-effects meta-analysis generated pooled odds ratios (ORs).

**Results:**

Seven reports representing six unique cohorts (6,825 patients) were included. Definitions used for early and late PTP timing varied across studies. Earlier PTP was associated with lower odds of VTE (pooled OR 0.47, 95% CI 0.23–0.99; I^2^ = 30.6%; *p* = 0.046). The association was of borderline statistical significance: it did not persist on leave-one-out removal of either large contributing cohort, and was non-significant when re-estimated as a risk ratio (RR 0.50, 95% CI 0.25–1.02; *p* = 0.057). No difference in in-hospital mortality was observed (pooled OR 1.15, 95% CI 0.90–1.46; *p* = 0.253). Evidence regarding haemorrhage progression was derived from a single cohort with three total events (OR 0.55, 95% CI 0.03–11.52; *p* = 0.701); assessment relied on qualitative binary radiological review, which is insensitive to small-to-moderate progression. Among patients receiving PTP, pooled VTE prevalence was 10.1% (95% CI 5.9–15.3%), varying nearly fourfold (5.5%-20.7%) according to surveillance intensity. Three cohorts were judged at moderate and three at serious risk of bias; certainty of evidence was very low for all primary outcomes.

**Conclusions:**

In adults with severe TBI (defined by GCS 8 or less in 100% of the analysed cohort), earlier PTP was associated with lower VTE risk of borderline statistical significance, and no difference in in-hospital mortality. The direction was consistent with prior mixed-severity syntheses, but the estimate was fragile under sensitivity analyses. The safety of earlier PTP with respect to intracranial haemorrhage progression remains unresolved, as only one cohort contributed this outcome using qualitative assessment. Adequately powered randomised evidence with quantitative haemorrhage assessment is warranted.

**Supplementary Information:**

The online version contains supplementary material available at https://doi.org/10.1007/s00701-026-07002-2.

## Introduction

Traumatic brain injury (TBI) affects an estimated 69 million people annually and accounts for approximately 30% of injury-related deaths worldwide, representing a leading cause of death and disability across all age groups [13, 51]. Severe TBI is defined by clinical and radiological criteria, including a Glasgow Coma Scale (GCS) of 8 or less, prolonged loss of consciousness, post-traumatic amnesia lasting more than seven days, and structural neuroimaging findings. Severe TBI accounts for a minority of presentations but a disproportionate share of mortality, long-term neurological disability, and neurocritical care utilisation [20, 28, 35]. Although severity classification incorporates multiple domains, GCS remains the principal bedside measure when classifying the severity of TBI into its three strata in acute neurocritical care. Survivors face protracted intensive care admissions requiring sedation, mechanical ventilation, and repeated interventions, increasing vulnerability to secondary complications beyond the primary brain injury [49].

Venous thromboembolism (VTE) is among the most consequential of these complications and its effects are compounded in severe TBI. Patients with severe TBI frequently face prolonged immobility, polytrauma, and associated injuries such as pelvic and long bone fractures. These patterns of injury potentiate venous stasis and endothelial damage creating a highly thrombogenic environment [29]. The clinical burden is substantial: deep vein thrombosis (DVT) develops in up to 54% of patients with major head injury in the absence of pharmacological thromboprophylaxis (PTP) [18]. Concurrently, severe TBI is commonly characterised by large intracranial haemorrhages with high propensity for expansion, where even small increases in volume may precipitate rapid neurological deterioration and death [9, 15]. PTP may reduce thromboembolic events but risks exacerbating intracranial haemorrhage in an unstable intracranial environment. This dilemma is present in all TBI but most pronounced in severe TBI, where both the thrombotic and haemorrhagic risks are highest.

The consequences of treatment failure amplify this tension. If prophylaxis is withheld and VTE occurs, management necessitates full-dose therapeutic anticoagulation in the setting of recent intracranial haemorrhage, a substantially higher-risk scenario than prophylactic anticoagulation [42]. Pulmonary embolism (PE) remains a major cause of preventable morbidity and mortality after trauma, and severe cases may require thrombolysis, catheter-directed intervention, or surgical embolectomy, each carrying significant bleeding risk [3, 26]. The timing of PTP represents a narrow therapeutic window in which both early and delayed initiation carry clinically meaningful risks.

These competing risks are not weighted symmetrically in clinical decision-making. Intracranial haemorrhagic complications associated with what may be deemed as “premature” anticoagulation are immediate and visible. Thromboembolic events emerge later and are less readily attributed to prophylaxis timing [2]. This omission bias is reinforced by interspecialty variation in risk prioritisation, with neurosurgical practice tending to emphasise haemorrhagic risk and critical care prioritising VTE prevention [6, 22]. These competing considerations contribute to substantial variation in practice.

Major international guidelines acknowledge this uncertainty but do not resolve it. The Brain Trauma Foundation acknowledges a role for PTP alongside mechanical measures but provides no timing recommendation due to insufficient evidence [8]. The Neurocritical Care Society recommends initiation within 24–48 h in patients with stable intracranial haemorrhage, based on low-quality evidence [34]. The UK National Institute for Health and Care Excellence (NICE) does not offer guidance on TBI specifically, stating only that PTP should be considered in major trauma patients or those undergoing cranial surgery when the risk of VTE outweighs the risk of intracranial haemorrhage [37]. The recent best practice guideline from the American College of Surgeons provides a dedicated chapter on timing of initiation by TBI severity, incorporating repeat computed tomography (CT) imaging, lesion-specific anatomical factors, and individual risk of haemorrhage progression [1]. This guidance reflects the desire to base challenging clinical decisions on iterative radiological and clinical information.

Eleven systematic reviews and meta-analyses have addressed the timing of PTP in TBI over the past decade, reporting reduced VTE with earlier initiation [4, 11, 16, 23, 24, 27, 30, 31, 45, 47, 52]. However, none has restricted its analysis to patients with severe TBI defined by the GCS criteria. Most include populations defined by Abbreviated Injury Scale (AIS), which does not capture neurological severity and may include patients with mild or moderate brain injury alongside critically ill cohorts. The most recent and largest review, by Karmur et al. [24], originally prespecified a GCS 8 or less severity criterion but broadened eligibility to accommodate AIS-defined cohorts, as the available literature was felt by the authors to be incompatible with the strict criterion. The present review therefore complements Karmur et al. by testing whether the pooled association observed under broader eligibility persists when analysis is restricted to the strict GCS 8 or less stratum. Furthermore, two published reports describing the same single-centre cohort have been treated as independent studies in prior meta-analyses, introducing double-counting of patient-weights that biases pooled estimates [12, 44]. Consequently, severity-specific evidence regarding the balance between thromboembolic benefit and haemorrhagic risk remains limited.

This systematic review and meta-analysis evaluates the effect of pharmacological thromboprophylaxis timing on VTE, intracranial haemorrhage progression, and mortality in adults with severe TBI, restricted to cohorts with a GCS score of 8 or less.

## Materials and methods

This systematic review and meta-analysis was conducted in accordance with the Preferred Reporting Items for Systematic Reviews and Meta-Analyses (PRISMA) 2020 statement [38]. The protocol was registered a priori on the International Prospective Register of Systematic Reviews (PROSPERO; registration number CRD420261388300).

### Search strategy and study selection

MEDLINE (via Ovid), EMBASE (via Ovid), and the Cochrane Central Register of Controlled Trials (CENTRAL) were searched from database inception to 1 May 2026. Search terms covered three domains: TBI, VTE, and PTP, combining exploded subject headings (MeSH for MEDLINE and CENTRAL; Emtree for EMBASE) with title and abstract free-text terms. The full search strategy for each database is provided in Supplementary Table 1. Searches were restricted to human studies in English. Three studies known a priori to meet the inclusion criteria (Byrne 2016, Hachem 2018, Skrifvars 2017) were used as sentinel checks; all three were retrieved. Database searches were supplemented by backward and forward citation searching of all included studies and of eleven existing systematic reviews on this topic. The PROSPERO register and Cochrane Database of Systematic Reviews were additionally searched to identify ongoing or recently completed reviews on this topic.

### Eligibility criteria

Eligible studies were original primary research of any observational or experimental design reporting on the timing or use of PTP in adults (≥ 16 years) with severe TBI. The principal methodological criterion was a strict GCS-based severity definition: a study was eligible only if 100% of the analysed cohort had a GCS score of 8 or less, or if raw event counts and timing data for a clearly defined GCS 8 or less subgroup were separately extractable from the published manuscript. Studies defining severity by anatomical surrogates alone, including head Abbreviated Injury Scale (AIS) ≥ 3 or ICD-coded severity, without explicit GCS-based confirmation, were excluded. Additional exclusion criteria were: non-human studies; non-English language; studies reporting only on therapeutic anticoagulation for established VTE rather than prophylaxis; rehabilitation-only populations; and studies of non-traumatic intracranial haemorrhage.

Title and abstract screening was performed independently by two reviewers using Rayyan [36], followed by full-text assessment for inclusion. Disagreements were resolved by consensus with a third reviewer.

### Outcomes

The primary outcome was the incidence of VTE, defined as a composite measure including DVT and/or PE during the index hospitalisation, compared between early and late initiation of PTP. Definitions of DVT, PE, and timing thresholds for prophylaxis were those used by the investigators in the individual studies. No fixed timepoint cutoff other than hospital discharge was applied. VTE-attributable mortality was not incorporated into the composite outcome as no contributing study reported this as a distinct endpoint. Secondary outcomes were progression of intracranial haemorrhage on follow-up imaging, reported as a binary outcome, and in-hospital all-cause mortality. Two additional analyses were prespecified: the comparative effect of PTP plus mechanical prophylaxis versus mechanical prophylaxis alone on VTE and mortality in cohorts that included a PTP-naive comparator group, and the pooled prevalence of VTE among patients receiving PTP across all contributing cohorts. PE events were also summarised descriptively, given inconsistent reporting across cohorts.

### Data extraction and quality assessment

Data were extracted independently by two reviewers onto a prespecified form covering study design, population characteristics, exposure classification, outcome definitions, event counts, and statistical methods. Discrepancies were resolved by discussion and, where necessary, involvement of a third reviewer. Risk of bias was assessed using the Risk of Bias in Non-Randomised Studies of Interventions tool (ROBINS-I) [48]. Certainty of evidence for each pooled outcome was rated using the Grading of Recommendations Assessment, Development and Evaluation (GRADE) framework [43]. As all included studies were observational, certainty ratings started at low and were subsequently modified according to established GRADE criteria.

### Statistical analysis

In accordance with the prespecified analysis plan, random-effects meta-analysis using the DerSimonian-Laird method was performed for outcomes with sufficient data. Dichotomous outcomes were summarised as odds ratios (ORs) with 95% confidence intervals (CIs), with the early PTP arm as the reference in all early-versus-late comparisons. Statistical heterogeneity was assessed using Cochran's Q test and quantified with the I^2^ statistic and τ^2^, with I^2^ interpreted on standard thresholds of 25%, 50%, and 75% as low, moderate, and high respectively [21]. Zero-cell tables were handled by Haldane's continuity correction, with the arcsine-difference effect measure as a sensitivity analysis. The pooled-proportion analysis used the Freeman-Tukey double-arcsine transformation [5]. Prespecified sensitivity analyses included leave-one-out re-estimation, substitution of the restricted maximum likelihood estimator for DerSimonian-Laird, and re-estimation using risk ratios instead of odds ratios. A DVT-only sensitivity analysis was additionally prespecified to account for the inconsistent reporting of PE across cohorts. Prespecified subgroup analyses examined risk of bias, VTE surveillance method and study design. All analyses were performed in R using the metafor package [41].

## Results

**Fig. 1:**
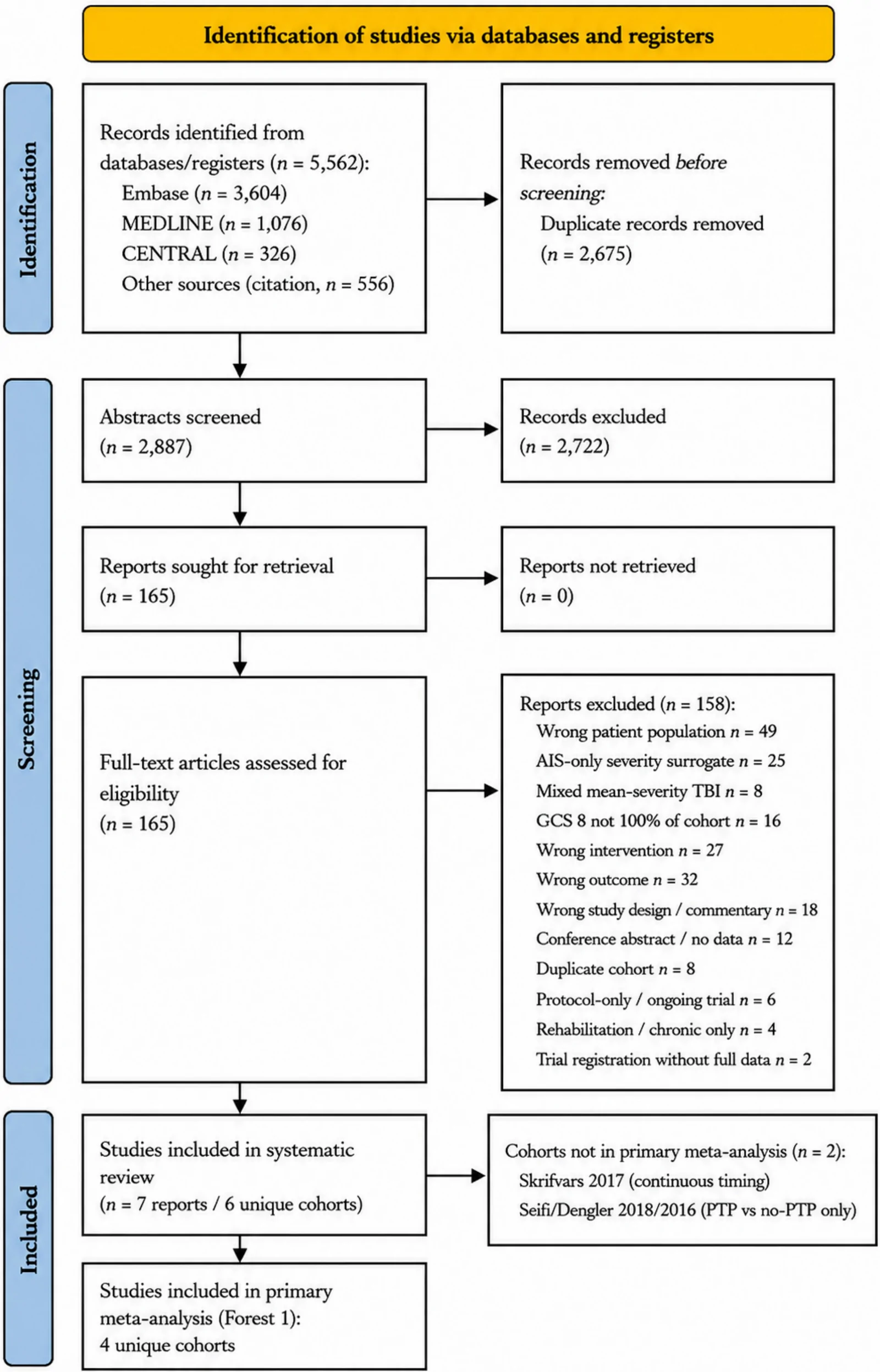
PRISMA flow diagram of study selection

A total of 5,562 records were identified, of which seven reports representing six unique cohorts met the inclusion criteria after deduplication, title and abstract screening, and full-text review [7, 12, 14, 19, 25, 44, 46] (Fig. 1). Inter-rater reliability for title and abstract screening was almost perfect (Cohen's kappa κ = 0.82). The principal reason for exclusion at full-text review was failure to meet the prespecified severe TBI criterion (GCS 8 or less). The included studies comprised three multicentre registry analyses, two single-centre cohort studies, and one post hoc analysis of a randomised trial. The reports by Seifi et al. and Dengler et al. described the same single-centre cohort and were therefore analysed as a single study [12, 44].

### Study characteristics

The six unique cohorts comprised 6,825 patients with severe TBI and were conducted between 2009 and 2021 across the United States, Canada, and a multinational consortium spanning Australia, New Zealand, Europe, and Saudi Arabia (Table 1). Four cohorts [7, 14, 19, 25] contributed to the primary meta-analysis of early versus late PTP initiation. Skrifvars et al. [46] and Seifi et al./Dengler et al. [12, 44] were included only in secondary analyses because they did not report binary early versus late comparisons. Byrne et al. [7] excluded patients with severe extracranial injuries (head AIS ≥ 3 in other body regions), restricting that cohort to predominantly isolated TBI. The remaining cohorts included patients with concomitant extracranial injuries without characterisation of injury type.

**Table 1:** Characteristics of included studies

Study	Country/Setting	Design	Period	N	Severity inclusion	Timing comparison	Prophylaxis agent(s)	VTE surveillance	Outcomes reported
Byrne 2016	USA + Canada (ACS-TQIP; 186 Level I/II trauma centres across North America)	Retrospective cohort with propensity-score matching	Jan 2012 – Dec 2014	3,634 (PSM-matched 2,468)	GCS ≤ 8 and head AIS ≥ 3	< 72 h vs ≥ 72 h	LMWH or UFH	Administrative TQIP coding; routine screening reported at 13% of contributing centres	DVT, PE, mortality, late craniotomy/intracranial monitor or drain placement
Elkbuli 2024	USA (796 trauma centres, ACS-TQIP)	Retrospective cohort (TQIP)	2017–2021	3,609	GCS ≤ 8 (explicit exclusion of GCS > 8) + head AIS > 2	≤ 24 h vs > 24 h	LMWH 74%/UFH 26%	Administrative TQIP coding; surveillance practice varies by centre	DVT, PE, mortality, craniotomy after PTP
Skrifvars 2017	Multinational EPO-TBI consortium: Australia, New Zealand, Finland, France, Ireland, Saudi Arabia	Post-hoc analysis of EPO-TBI RCT	May 2010 – Nov 2014	454 (severe-TBI subgroup of 603 EPO-TBI cohort)	GCS 3–8 (severe stratum only)	Continuous time-dependent (Cox); no binary dichotomy	Mechanical in 91–98% across the ICU stay; pharmacological in 75% (typically enoxaparin 40 mg SC daily)	Twice-weekly bilateral compression Doppler US (proximal-only)	DVT, PE, composite VTE with overlap, mortality
Seifi/Dengler 2018/2016	USA (UTHSCSA Neuro-ICU, San Antonio TX — single centre)	Retrospective single-centre cohort	Jan 2011 – Dec 2013	155 (PTP 122/no-PTP 33)	GCS < 8 (Dengler); GCS ≤ 8 (Seifi). The stricter Dengler definition was applied throughout	PTP vs no-PTP (no early/late)	UFH 5,000 U SC BD/TDS; LMWH 30 mg SC BD or 40 mg SC daily	Symptomatic-trigger only	DVT (Dengler), PE (Seifi), ICH progression (Dengler); mortality reported identically in both papers
Kim L 2014	USA (Hospital of the University of Pennsylvania, Philadelphia — single centre)	Retrospective chart review	Jan 2009 – Dec 2010	75 (early 22/intermediate 34/late 19)	GCS ≤ 8 + head AIS ≥ 3 + ISS ≥ 11 + LOS > 3 d	< 72 h vs 72–120 h vs > 5 d	UFH TDS or LMWH BD	Symptomatic-trigger extremity duplex US (DVT) + CT-PA or V/Q scan (PE)	DVT, PE, mortality, ICH-progression trajectory score
Hachem 2018	Canada (Sunnybrook Health Sciences Centre, Toronto — single centre)	Prospective observational cohort	Oct 2015 – Sep 2016	64	GCS ≤ 8 (consecutive enrolment)	< 3 d vs ≥ 3 d vs no-PTP	Enoxaparin (weight-adjusted SC; 94% received 40 mg daily); TED stockings applied to all patients on admission	Routine duplex US day 7 (± 3) + symptomatic-trigger	DVT, PE, DVT + PE (mutually exclusive), ICH progression, mortality

Abbreviations: *ACS-TQIP* American College of Surgeons Trauma Quality Improvement Program, *AIS* Abbreviated Injury Scale, *BD* twice daily, *CT-PA* computed tomography pulmonary angiography, *DVT* deep vein thrombosis, *EPO-TBI* Erythropoietin in Traumatic Brain Injury, *GCS* Glasgow Coma Scale, *ICH* intracranial haemorrhage, *ICU* intensive care unit, *ISS* Injury Severity Score, *LMWH* low molecular weight heparin, *LOS* length of stay, *PE* pulmonary embolism, *PSM* propensity score matched, *PTP* pharmacological thromboprophylaxis, *RCT* randomised controlled trial, *SC* subcutaneous, *TDS* three times daily, *TED* thromboembolic deterrent stockings, *UFH* unfractionated heparin, *US* ultrasonography, *UTHSCSA* University of Texas Health Science Center at San Antonio, *V/Q* ventilation/perfusion, *VTE* venous thromboembolism

Definitions of PTP timing varied across studies: Elkbuli et al. [14] defined early initiation as 24 h or less and late as more than 24 h after admission; Byrne et al. [7] and Hachem et al. [19] defined early as less than 72 h and late as 72 h or more; and Kim et al. [25] defined early as 72 h or less, intermediate as 72 to 120 h, and late as more than 120 h. Skrifvars et al. [46] modelled time to prophylaxis continuously by Cox regression, whereas the Seifi et al./Dengler et al. [12, 44] cohort compared PTP with no PTP. Intracranial haemorrhage progression was assessed by qualitative binary radiological assessment in Hachem et al. [19] and Seifi et al./Dengler et al. [12, 44], by graded trajectory score in Kim et al. [25], and by neurosurgical intervention rate as an indirect proxy in Byrne et al. [7] and Elkbuli et al. [14]. VTE ascertainment also differed across studies: four cohorts relied on clinically indicated investigations [7, 12, 14, 25, 44], one used protocolised duplex ultrasonography on day 7 of admission [19], and one performed twice-weekly screening duplex ultrasonography throughout the intensive care unit stay [46]. The timing of individual VTE events within the admission was not consistently reported across contributing cohorts [7, 14, 19, 25]. Mechanical prophylaxis, comprising thromboembolic deterrent stockings or sequential compression devices, was used across all patients in Hachem et al. [19], Seifi et al./Dengler et al. [12, 44], and Byrne et al. [7], and in 91–98% of patients in Skrifvars et al. [46]; use was not documented in Elkbuli et al. [14] or Kim et al. [25].

### Venous thromboembolism

**Fig. 2:**
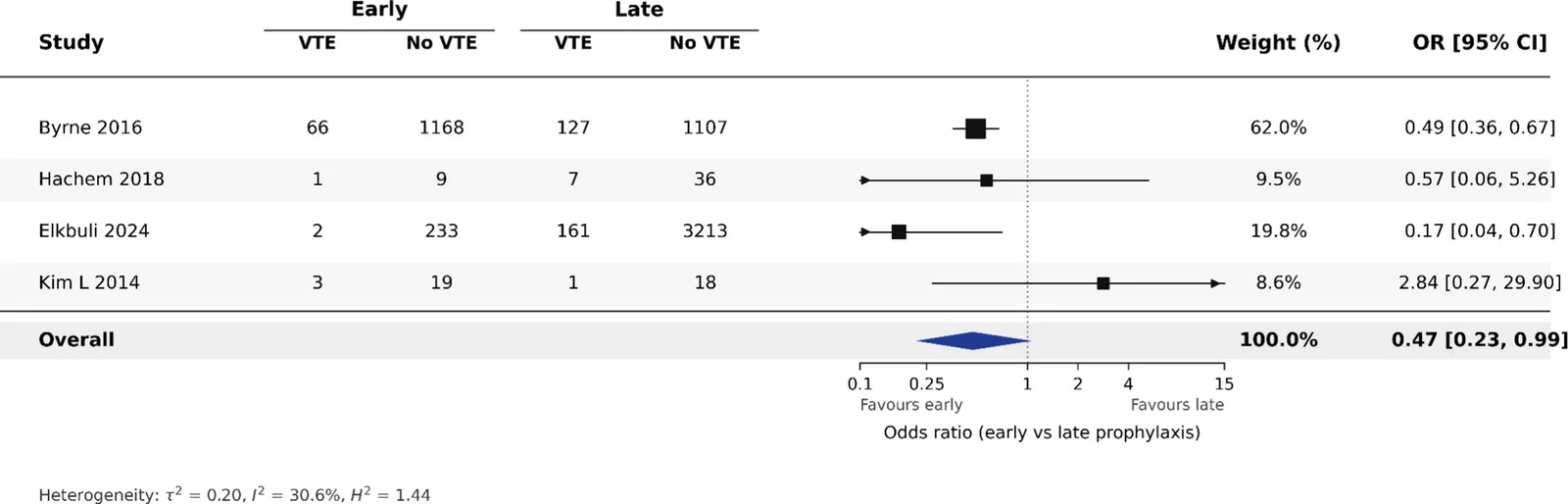
Forest plot of VTE incidence comparing early versus late pharmacological thromboprophylaxis in adults with severe TBI. Individual study odds ratios and 95% confidence intervals are shown; the diamond represents the pooled random-effects estimate. Abbreviations: CI = confidence interval; OR = odds ratio; VTE = venous thromboembolism; TBI = traumatic brain injury

Four cohorts [7, 14, 19, 25] contributed to the primary analysis of early versus late PTP initiation (*n* = 6171; early *n* = 1501, late *n* = 4670). Early PTP was associated with lower odds of VTE than late PTP (pooled OR 0.47, 95% CI 0.23 to 0.99; I^2^ = 30.6%; *p* = 0.046; Fig. 2). The direction of effect was consistent across three of four cohorts [7, 14, 19]. A prespecified DVT-only sensitivity analysis (*n* = 6171) returned a concordant estimate (pooled OR 0.50, 95% CI 0.28 to 0.92; I^2^ = 17.5%; *p* = 0.026; Supplementary Fig. 1).

Two cohorts [12, 19, 44] contributed to the comparison of PTP plus mechanical prophylaxis versus mechanical prophylaxis alone (*n* = 219; PTP + mechanical *n* = 175, mechanical alone *n* = 44). No significant difference in VTE incidence was observed between PTP plus mechanical prophylaxis and mechanical prophylaxis alone (OR 0.32, 95% CI 0.08 to 1.32; I^2^ = 53.0%; *p* = 0.116; Supplementary Fig. 2).

**Fig. 3:**
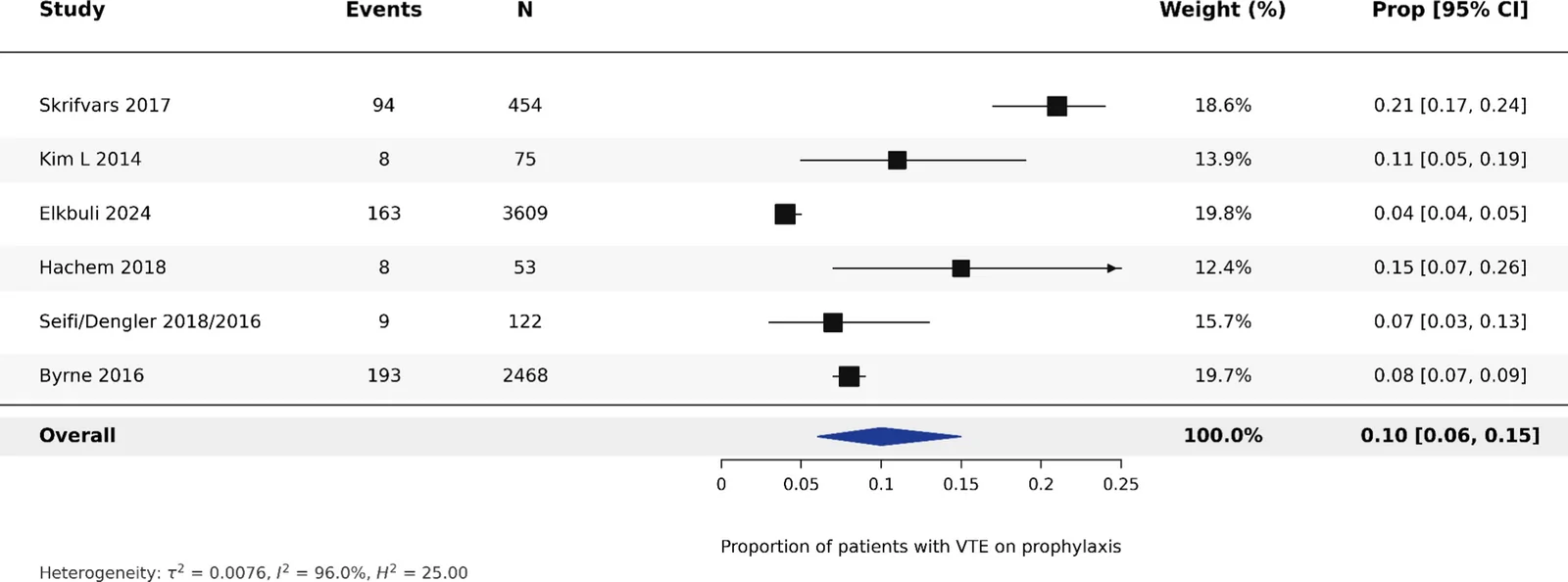
Forest plot of pooled VTE prevalence among patients receiving pharmacological thromboprophylaxis across all six contributing cohorts. Individual study proportions and 95% confidence intervals are shown; the diamond represents the pooled random-effects estimate Abbreviations: CI = confidence interval; Prop = proportion; VTE = venous thromboembolism

All six cohorts contributed to the pooled prevalence analysis of VTE among patients receiving PTP (*n* = 6,781). The pooled prevalence was 10.1% (95% CI 5.9 to 15.3%; I^2^ = 96%; Fig. 3). Prevalence estimates varied according to surveillance strategy, ranging from 5.5% in cohorts using clinically indicated investigations [7, 12, 14, 25, 44] to 15.1% in the cohort applying protocolised day-7 duplex ultrasonography [19] and 20.7% in the cohort using twice-weekly screening ultrasonography [46].

### Intracranial haemorrhage progression

One cohort [19] reported intracranial haemorrhage progression as a binary outcome according to PTP timing. Progression occurred in 0 of 10 patients receiving early PTP (0%) and 3 of 43 receiving late PTP (7.0%) (OR 0.55, 95% CI 0.03 to 11.52; *p* = 0.701). In the PTP versus no PTP comparison, reported in one cohort [12, 44], progression was recorded in 12 of 122 (9.8%) patients receiving prophylaxis and 0 of 33 (0%) receiving none. Rates of neurosurgical intervention after PTP initiation were similar between groups: Byrne et al. [7] reported late craniotomy or craniectomy in 2.5% versus 2.9% (adjusted OR 0.86, 95% CI 0.53 to 1.40) and Elkbuli et al. [14] reported corresponding rates of 3.4% versus 2.9% (*P* = 0.79).

### Mortality

**Fig. 4:**
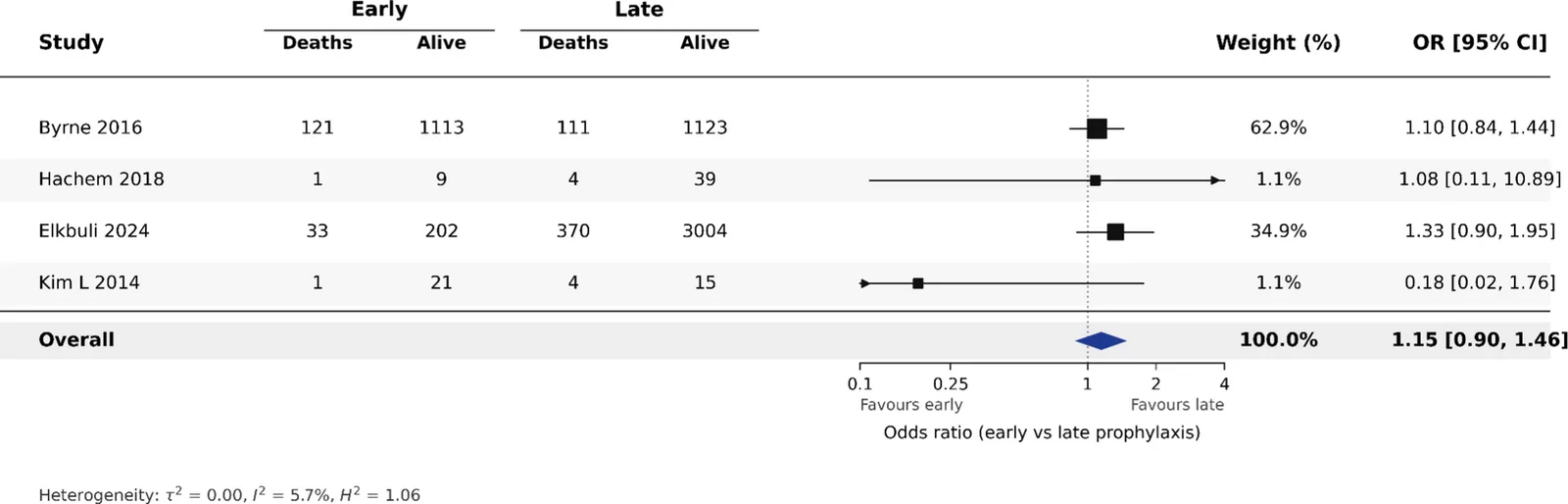
Forest plot of in-hospital mortality comparing early versus late pharmacological thromboprophylaxis in adults with severe TBI. Individual study odds ratios and 95% confidence intervals are shown; the diamond represents the pooled random-effects estimate. Abbreviations: CI = confidence interval; OR = odds ratio; TBI = traumatic brain injury

Four cohorts [7, 14, 19, 25] contributed to the meta-analysis of in-hospital mortality (*n* = 6171; early *n* = 1501, late *n* = 4670). No significant difference was observed between early and late PTP initiation (pooled OR 1.15; 95% CI 0.90 to 1.46; I^2^ = 5.7%; *p* = 0.253; Fig. 4). Unadjusted mortality estimates were consistent across all four cohorts. Elkbuli et al. [14] reported higher mortality with early PTP on multivariable adjustment (adjusted OR, 1.61; 95% CI, 1.06 to 2.50), a finding not apparent in the unadjusted estimate shown in Fig. 4 (OR 1.33; 95% CI 0.90 to 1.95).

### Sensitivity and subgroup analyses

Sensitivity analyses were consistent with the primary findings. For VTE, the pooled estimate remained below 1.0 in all leave-one-out analyses (range OR 0.46 to 0.53), though no significant association was observed after exclusion of either of the two largest contributing cohorts [7, 14]. Use of a restricted maximum-likelihood estimator yielded a statistically significant association (OR 0.51; 95% CI 0.36 to 0.70; *p* < 0.001). Re-estimation using risk ratios did not reach statistical significance (RR 0.50; 95% CI 0.25 to 1.02; *p* = 0.057). For intracranial haemorrhage progression, the arcsine-difference analysis produced a concordant estimate (AS − 0.27; SE, 0.18). For mortality, no significant difference was observed across any leave-one-out iteration (range OR 0.94 to 1.17).

Prespecified subgroup analyses were limited by the small number of contributing studies. Variation in VTE surveillance strategy appeared to contribute substantially to heterogeneity in pooled prevalence estimates.

### Risk of bias and certainty of evidence

**Fig. 5:**
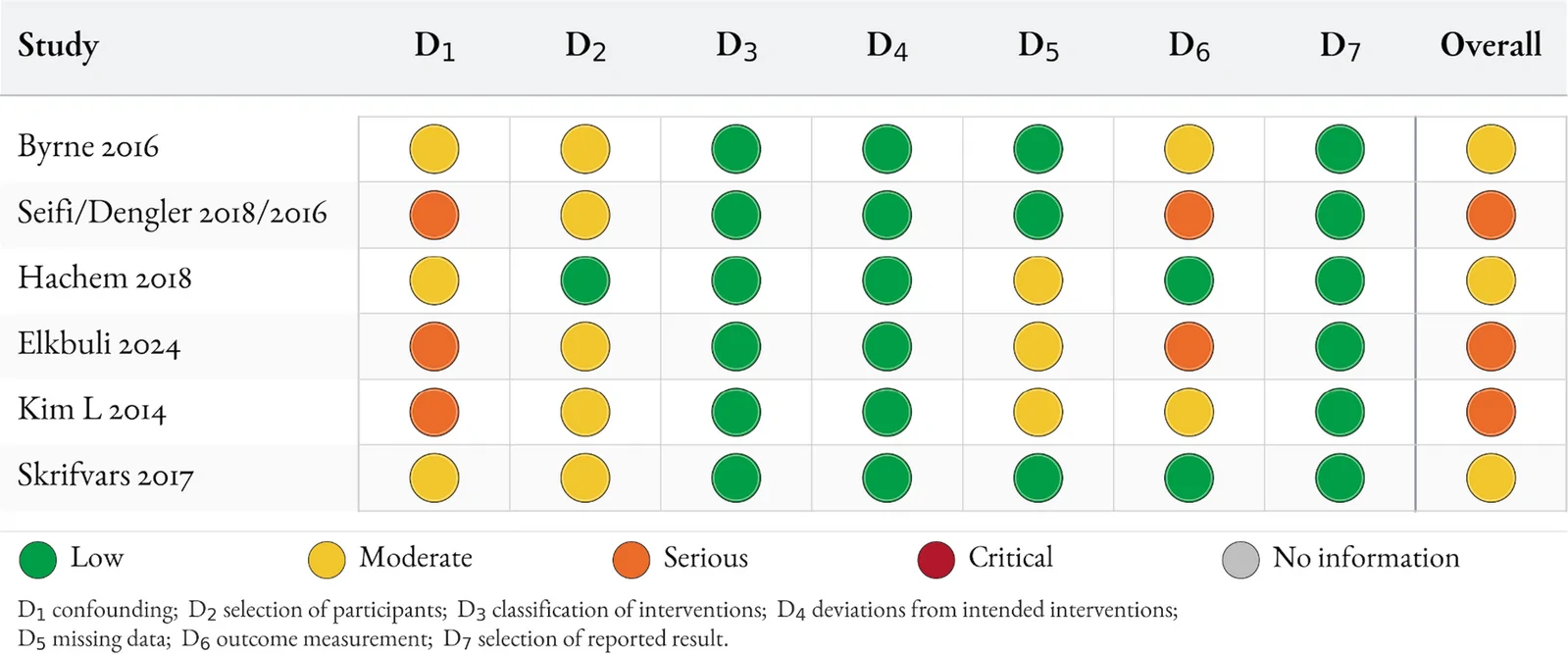
ROBINS-I risk of bias assessment for each included cohort

Three cohorts [7, 19, 46] were judged to be at moderate overall risk of bias on ROBINS-I assessment, whereas the remaining three [12, 14, 25, 44] were judged to be at serious risk of bias, driven principally by confounding by indication and baseline imbalances between treatment groups (Fig. 5). Certainty of evidence was rated very low for all primary outcomes (Table 2), reflecting observational study designs, indirectness arising from heterogeneous prophylaxis-timing definitions, imprecision in smaller analyses, and suspected publication bias.

**Table 2:** GRADE evidence profile for primary outcomes

		Certainty assessment					Number of patients		Effect		Certainty	Importance
№ studies	Study design	Risk of bias	Inconsistency	Indirectness	Imprecision	Other considerations	Early/PTP + mechanical	Late/Mechanical alone	Relative (95% CI)	Absolute (95% CI)		
Venous thromboembolism — Early vs late pharmacological prophylaxis												
4	Non-randomised studies (observational cohorts)	Serious^a^	Not serious	Serious^c^	Not serious	Publication bias suspected^d^	72/1,501 (4.8%)	296/4,670 (6.3%)	**OR 0.47** (0.23 to 0.99)	**32 fewer per 1,000** (from 48 to 1 fewer)	** ⊕ ○○○ Very low**^a,c,d^	Important
Venous thromboembolism — Pharmacological plus mechanical prophylaxis vs mechanical prophylaxis alone												
2	Non-randomised studies	Serious^a^	Cannot assess^e^	Not serious	Very serious^b^	Cannot assess^c^	17/175 (9.7%)	12/44 (27.3%)	**OR 0.32** (0.08 to 1.32)	**166 fewer per 1,000** (from 244 fewer to 58 more)	** ⊕ ○○○ Very low**^a,b,e^	Important
Intracranial haemorrhage progression — Early vs late pharmacological prophylaxis												
1	Non-randomised study (Hachem 2018)	Not serious	Cannot assess^f^	Serious^g^	Very serious^b^	Cannot assess^f^	0/10 (0%)	3/43 (7.0%)	**OR 0.55** (0.03 to 11.52)	**30 fewer per 1,000**^i^ (from 68 fewer to 394 more)	** ⊕ ○○○ Very low**^b,f,g^	Critical
Mortality — Early vs late pharmacological prophylaxis												
4	Non-randomised studies	Serious^a^	Serious^h^	Serious^c^	Not serious	Publication bias suspected^d^	156/1,501 (10.4%)	489/4,670 (10.5%)	**OR 1.15** (0.90 to 1.46)	**14 more per 1,000** (from 9 fewer to 41 more)	** ⊕ ○○○ Very low**^a,c,d,h^	Critical

^a^ ≥ 2 cohorts at serious ROBINS-I risk of bias

^b^ Wide 95% CI and/or crosses no effect

^c^ Variable early/late prophylaxis definitions

^d^ Publication bias suspected (< 10 studies)

^e^ Inconsistency not assessable (2 studies)

^f^ Inconsistency not assessable (1 study)

^g^ Binary ICH progression outcome (non-volumetric)

^h^ Heterogeneity in mortality estimates

^i^ Derived from upper 95% CI of relative effect

Abbreviations: *CI* confidence interval, *GRADE* Grading of Recommendations Assessment, Development and Evaluation, *OR* odds ratio, *PTP* pharmacological thromboprophylaxis, *VTE* venous thromboembolism

## Discussion

### Principal findings

Across six cohorts comprising 6,825 patients with severe TBI and GCS 8 or less, earlier PTP, as defined by the individual studies, was associated with lower odds of VTE than later initiation (pooled OR 0.47, 95% CI 0.23 to 0.99). This finding is consistent with pooled estimates from eleven prior systematic reviews [4, 11, 16, 23, 24, 27, 30, 31, 45, 47, 52] ranging from an OR of 0.38 to 0.58. No association was observed for in-hospital mortality. Evidence regarding haemorrhagic safety was limited, with only a single cohort [19] contributing directly comparable data and overall certainty rated very low. These findings suggest that delayed PTP may carry a measurable thromboembolic cost in severe TBI though safety of early PTP with regard to haemorrhagic progression remains uncertain.

### Earlier prophylaxis and venous thromboembolism risk

Earlier initiation of PTP was associated with approximately half the odds of VTE compared with later PTP (pooled OR 0.47, 95% CI 0.23 to 0.99), a direction of effect preserved across all prespecified sensitivity analyses. Despite this association, the absolute burden of VTE remained substantial, with one in ten patients receiving PTP experiencing a VTE event.

The pooled estimate should be read with an important structural caveat: Byrne et al. [7] contributes approximately 62% of the meta-analytic weight. The pooled result therefore reflects a single propensity-matched registry cohort, with the three smaller contributing cohorts providing directional corroboration rather than independent statistical support. Consistent with this weighting, statistical significance was lost on exclusion of either of the two large registry cohorts [7, 14], and risk ratio substitution yielded an upper confidence interval bound marginally crossing the null.

The estimate derives exclusively from observational data and remains susceptible to residual confounding, particularly confounding by indication. Patients selected for delayed PTP often have unstable intracranial haemorrhage on serial CT, larger haemorrhage burden, intracranial hypertension, coagulopathy, or require ongoing neurosurgical intervention. These clinical characteristics both influence PTP timing and independently increase the risk of VTE and adverse outcomes. Consequently, the observed association may partially reflect differences in underlying injury severity rather than the timing of PTP. The findings are therefore most appropriately interpreted as evidence of a consistent association rather than proof of a causal treatment effect. A secondary analysis restricted to cohorts including a no PTP comparator, in which mechanical prophylaxis was applied across both arms, similarly did not demonstrate a significant incremental benefit from PTP (OR 0.32, 95% CI 0.08 to 1.32), though this comparison was limited by small event numbers, wide confidence intervals, and substantial heterogeneity between the two contributing cohorts.

The absence of an association between PTP timing and in-hospital mortality (pooled OR 1.15, 95% CI 0.90 to 1.46; I^2^ = 5.7%; *p* = 0.253) should be interpreted cautiously. Mortality is a relatively insensitive outcome in severe TBI, where survival is determined predominantly by injury severity and neurological trajectory rather than modest differences in PTP timing. Several mechanisms may account for absence of a mortality difference despite the observed reduction in VTE. Any survival benefit from fewer VTE events may be offset by competing causes of death, while prompt diagnosis and treatment of VTE may attenuate its impact on mortality. Furthermore, the in-hospital follow-up of the included studies may be insufficient to capture the longer-term mortality associated with VTE. Among patients hospitalised with DVT, all-cause mortality increases from approximately 5% at 30 days to nearly 20% at one year [32], suggesting that clinically meaningful survival differences may emerge beyond the observation periods of these studies. Although one cohort [14] reported higher adjusted mortality with earlier PTP, this estimate is difficult to interpret given marked baseline imbalances, including a 14:1 arm asymmetry, greater injury severity in the late PTP group, and differences in PTP agent allocation. Paradoxically, the late prophylaxis group demonstrated lower mortality despite greater baseline severity, suggesting substantial residual confounding rather than a true treatment effect. Consequently, the absence of a mortality signal should not be interpreted as evidence that PTP timing has no clinically meaningful effect on patient outcomes.

Across eleven prior reviews of heterogeneous TBI populations (none restricted exclusively to GCS 8 or less) [4, 11, 16, 23, 24, 27, 30, 31, 45, 47, 52], pooled VTE odds ratios have ranged from 0.38 to 0.58, with the present estimate of 0.47 lying within this range. Karmur et al. [24], the largest and most recent review on this topic enrolling 24,401 patients across fourteen studies, reported an identical pooled odds ratio (0.47) despite employing substantially broader eligibility criteria. The convergence of two independent syntheses on the same pooled point estimate, under different inclusion definitions, is best interpreted as external validation of the association rather than as an independent finding. The present review therefore does not establish a new effect estimate. It confirms the direction and magnitude of the existing evidence under a stricter severity definition, and adds three specific methodological refinements: (i) a severity-specific denominator that directly informs the severe-TBI subgroup analysis planned in the ongoing TOP-TBI trial; (ii) resolution of the Seifi 2018/Dengler 2016 dual-report cohort overlap that has been treated as two independent studies in prior meta-analyses including Karmur et al.; and (iii) a surveillance-intensity stratified analysis of pooled VTE prevalence, which explains the apparent between-study variation as a measurement artefact rather than a true prevalence range.

### Intracranial haemorrhage progression: a critical evidence gap

The haemorrhagic safety of earlier PTP in severe TBI remains uncertain. Only one cohort [19] reported binary intracranial haemorrhage progression according to PTP timing, assessed by qualitative radiological review, with three events among 53 patients (0 of 10 in the early group versus 3 of 43 in the late group; OR 0.55, 95% CI 0.03 to 11.52). Although the point estimate favoured earlier PTP, the wide confidence interval precludes any firm inference.

Nevertheless, the available evidence provides no clear signal that earlier PTP increases intracranial haemorrhagic risk. Kim et al. [25] observed the slowest trajectory of cumulative haemorrhage progression in the early PTP arm, while late neurosurgical intervention rates were comparable between treatment groups in the two largest cohorts [7, 14]. Two recent meta-analyses conducted in broader TBI populations reported null associations between PTP timing and haemorrhage progression (Dengler et al. [11]: OR 0.79, 95% CI 0.56 to 1.12; Karmur et al. [24]: OR 1.01, 95% CI 0.79 to 1.29). One cohort [12, 44] recorded more haemorrhage progression among patients receiving PTP than among those receiving none; however, this comparison was substantially confounded by indication, with the no PTP arm substantially older (mean age 62 versus 40 years) and PTP withheld from patients perceived to be at highest haemorrhagic risk. Taken together, the directional consistency of the available evidence is reassuring, but falls short of establishing haemorrhagic safety.

The prevailing narrative of haemorrhagic safety rests on outcome measures that may be insufficiently sensitive to detect clinically meaningful progression. All included studies relied on qualitative binary radiological assessment rather than volumetric quantification, an approach that is inherently insensitive to small or moderate changes in haemorrhage volume. Automated segmentation methods now permit continuous, lesion-specific volumetric assessment on serial CT imaging and have demonstrated greater sensitivity for detecting haemorrhage progression [33]. Furthermore, no study accounted for variation in the interval between baseline and follow-up imaging. If patients receiving later PTP underwent imaging further from baseline, they had proportionally longer opportunities for progression to accumulate and be detected, introducing potential ascertainment bias. The apparent absence of a haemorrhagic signal may therefore reflect limitations of measurement rather than true biological safety.

### Surveillance intensity and VTE ascertainment

The observed burden of VTE varied markedly according to surveillance strategy, with a dose–response relationship between ascertainment intensity and apparent prevalence. Pooled VTE prevalence was 5.5% across the four cohorts using clinically indicated investigations [7, 12, 14, 25, 44], 15.1% in the single cohort applying protocolised duplex ultrasonography on day 7 of admission [19], and 20.7% in the cohort employing twice-weekly screening ultrasonography throughout the intensive care unit stay [46]. This finding is consistent with prior literature, demonstrating surveillance intensity is likely to be a major determinant of apparent VTE burden [10].

The clinical significance of this surveillance-detected excess remains uncertain. DVT was the strongest independent predictor of PE in a large administrative analysis of more than 200,000 patients with TBI (OR 9.85, 95% CI 5.5 to 17.6) [17], highlighting the potential for clinically significant downstream sequelae. However, routine ultrasonographic screening consistently increases DVT detection without a corresponding reduction in PE incidence [50], suggesting that detection and clinical consequence are not synonymous. Whether this reflects reporting accuracy, biological differences between screening-detected and symptomatic thrombi, or limitations in the management of asymptomatic thrombosis in an anticoagulation-averse population, remains unclear. Symptom-triggered investigation and protocolised screening therefore quantify different phenomena, and combining these surveillance paradigms into a single pooled prevalence estimate risks obscuring both.

### Clinical implications

Earlier PTP was consistently associated with lower VTE risk, while no difference in in-hospital mortality was observed. The pooled estimate was derived from studies using widely differing early-versus-late thresholds, from > 24 h in Elkbuli et al. [14] to > 120 h in Kim et al. [25], and therefore does not correspond to any single timing threshold. The direction of the association was, however, consistent across this spread of definitions, aligning with existing guideline recommendations that PTP should be initiated within 24 to 48 h of injury, once haemorrhage stability has been established [1, 34]. This synthesis does not identify an optimal timing window within that range. The finding should not be interpreted as evidence of universal safety with earlier PTP. Existing null findings for intracranial haemorrhage progression may reflect limitations of outcome ascertainment as much as biological safety.

Risk-stratification tools such as the modified Berne–Norwood criteria provide a structured framework for individualised decision-making by categorising patients according to lesion characteristics and estimated progression risk [39]. Similarly, the DEEP I trial demonstrated the safety of enoxaparin initiated within 24 h in selected low-risk intracranial haemorrhage patterns with stable imaging [40], but its findings cannot be extrapolated directly to the severe TBI population examined here.

The persistence of substantial practice variation despite institutional guidelines reflects the clinical uncertainty that observational evidence alone cannot resolve [6]. Concerns regarding haemorrhage progression and multidisciplinary disagreement continue to drive prophylaxis delay and inconsistent adherence to guidelines [6]. Randomised evidence in patients with severe TBI is therefore required to determine whether the apparent thromboembolic benefit of earlier PTP can be achieved without an accompanying haemorrhagic cost.

### Strengths and limitations

This review has several strengths. Eligibility was restricted to cohorts in which all analysed patients had severe TBI (GCS 8 or less). This represents the most stringent and clinically relevant severity definition applied when compared to previous reviews of this topic. This ensures direct applicability to the population in whom the timing of PTP is most consequential. Overlapping reports from the same cohort were identified and analysed as a single study [12, 44], avoiding double-counting of participants. The review was prospectively registered on PROSPERO and conducted in accordance with PRISMA 2020 standards. Risk of bias and certainty of evidence were assessed using ROBINS-I and GRADE. The direction of the pooled VTE effect remained consistent across all sensitivity analyses, despite the limited evidence base.

Nonetheless, several limitations warrant consideration. First, the evidence base was small and comprised exclusively observational studies, three of which were judged at serious overall risk of bias on ROBINS-I assessment [12, 14, 25, 44], with confounding by indication the principal concern across cohorts. As discussed above, the primary VTE estimate rested on a single dominant cohort (Byrne 2016), with the remaining cohorts providing directional support. Second, PTP timing definitions varied substantially across studies. For example, Elkbuli et al. [14] defined their late arm as PTP after 24 h whereas Kim et al. [25] defined their late arm as PTP after 120 h. This introduces serious indirectness and limits translation of the pooled estimate to any specific clinical threshold. Third, evidence on intracranial haemorrhage progression was sparse, with only a single cohort [19] contributing binary early versus late outcome data; assessment relied on qualitative radiological interpretation without volumetric quantification or adjustment for the interval between baseline and follow-up imaging. Fourth, although Byrne et al. [7] excluded patients with severe extracranial injuries, the remaining cohorts did not systematically characterise concomitant injury profiles. Whether the risk–benefit balance of earlier PTP differs in patients with pelvic fractures, solid organ lacerations, or other high-risk extracranial injuries therefore remains unaddressed. Finally, publication bias could not be formally assessed given the small number of contributing studies. Certainty of evidence was rated very low across all primary outcomes, and the findings of this review should not alone support strong clinical recommendations.

## Future directions

The principal priority for future research is high-quality randomised evidence defining the optimal timing of PTP in severe TBI. Although observational studies consistently suggest lower VTE risk with earlier PTP, residual confounding precludes causal inference. The ongoing TOP-TBI trial (ISRCTN64696029), which includes a prespecified severe TBI subgroup analysis, and the Canadian PROTEST trial (NCT03559114), a Bayesian adaptive randomised trial restricted exclusively to severe TBI, are expected to provide the first randomised evidence directly addressing this question. Future studies should adopt standardised definitions of PTP timing, VTE ascertainment, and intracranial haemorrhage progression to improve comparability across cohorts. Quantitative radiographic measures of haemorrhage progression may provide a more sensitive assessment of haemorrhagic safety than the qualitative approaches used to date. Prospective studies incorporating protocolised VTE surveillance may help define the true burden of VTE in this population, independent of ascertainment intensity. Finally, individual patient data meta-analysis pooling raw data across existing cohorts would increase statistical precision and permit more rigorous adjustment for confounding than possible through aggregate data synthesis alone.

## Conclusion

Earlier PTP was associated with lower VTE risk in adults with severe TBI (pooled OR 0.47, 95% CI 0.23–0.99), with no observed difference in in-hospital mortality. The VTE association was of borderline statistical significance, not persisting on leave-one-out removal of either large cohort or under risk-ratio re-estimation, and is best interpreted as directionally consistent rather than a robust effect. This confirms, under a strict severity definition (GCS 8 or less in 100% of the analysed cohort), the direction and magnitude of the association previously reported in mixed-severity syntheses. Because of heterogeneous timing definitions across the included studies, the pooled estimate does not identify an optimal timing window. The safety of earlier PTP with respect to intracranial haemorrhage progression also remains unresolved, as only one cohort contributed this outcome and assessment was qualitative. Whether the thromboembolic benefit of earlier PTP can be achieved without a haemorrhagic penalty requires adequately powered randomised evidence with quantitative haemorrhage assessment and standardised VTE ascertainment.

## Supplementary Information

Below is the link to the electronic supplementary material.

Supplementary file1 (DOCX 1437 KB)

## Data Availability

No datasets were generated or analysed during the current study.

## References

[1] American College of Surgeons (2024) Best practices in the management of traumatic brain injury. American College of Surgeons

[2] Awanzo A, Thompson J (2025) Cognitive biases in clinical decision-making in prehospital critical care; a scoping review. Scand J Trauma Resusc Emerg Med 33(1):101 10.1186/s13049-025-01415-1 PMC12135565 40462148

[3] Bahloul M, Chaari A, Dammak H et al (2011) Post-traumatic pulmonary embolism in the intensive care unit. Ann Thorac Med 6(4):199–206 10.4103/1817-1737.84773 PMC3183636 21977064

[4] Banerjee O, Rodrigues R, Adkins L, Busl KM (2025) Venous thromboembolism prophylaxis in the neurocritically ill population. J Clin Med 14(13):4434 10.3390/jcm14134434 PMC12249765 40648808

[5] Barendregt JJ, Doi SA, Lee YY, Norman RE, Vos T (2013) Meta-analysis of prevalence. J Epidemiol Community Health 67(11):974–978 10.1136/jech-2013-203104 23963506

[6] Burczak KW, Van Gent J-M, Bavishi D et al (2025) Variation exists in venous thromboembolism prophylaxis in traumatic brain injury despite national guidelines: insights from a recent AAST survey study. Trauma Surg Acute Care Open 10(4):e001708 10.1136/tsaco-2024-001708 PMC12557725 41158729

[7] Byrne JP, Mason SA, Gomez D, Hoeft C, Subacius H, Xiong W, Neal M, Pirouzmand F, Nathens AB (2016) Timing of pharmacologic venous thromboembolism prophylaxis in severe traumatic brain injury: a propensity-matched cohort study. J Am Coll Surg 223(4):621-631.e5 10.1016/j.jamcollsurg.2016.06.382 27453296

[8] Carney N, Totten AM, O’Reilly C et al (2017) Guidelines for the management of severe traumatic brain injury, Fourth Edition. Neurosurgery 80(1):6–15 10.1227/NEU.0000000000001432 27654000

[9] Cepeda S, Gómez PA, Castaño-Leon AM, Martínez-Pérez R, Munarriz PM, Lagares A (2015) Traumatic intracerebral hemorrhage: risk factors associated with progression. J Neurotrauma 32(16):1246–1253 10.1089/neu.2014.3808 25752340

[10] De Martino RR, Beck AW, Edwards MS, Corriere MA, Wallaert JB, Stone DH, Cronenwett JL, Goodney PP (2012) Impact of screening versus symptomatic measurement of deep vein thrombosis in a national quality improvement registry. J Vasc Surg 56(4):1045–51.e1 10.1016/j.jvs.2012.02.066 PMC3767393 22832263

[11] Dengler BA, Haight T, Fu A, Hafeez SJ, Cirivello M, Bartanusz V (2025) Safety of early chemoprophylaxis for venous thromboembolism after traumatic brain injury: a systematic review and meta-analysis. A military traumatic brain injury initiative study. J Neurosurg 142(4):1046–1057 10.3171/2024.7.JNS232697 39576983

[12] Dengler BA, Mendez-Gomez P, Chavez A, Avila L, Michalek J, Hernandez B, Grandhi R, Seifi A (2016) Safety of chemical DVT prophylaxis in severe traumatic brain injury with invasive monitoring devices. Neurocrit Care 25(2):215–223 10.1007/s12028-016-0280-8 27350548

[13] Dewan MC, Rattani A, Gupta S et al (2019) Estimating the global incidence of traumatic brain injury. J Neurosurg 130(4):1080–1097 10.3171/2017.10.JNS17352 29701556

[14] Elkbuli A, Watts E, Patel H, Chin B, Wright D-D, Inouye M, Nunez D, Rhodes HX (2024) National analysis of outcomes for adult trauma patients with isolated severe blunt traumatic brain injury following venous thromboembolism prophylaxis. J Surg Res 300:165–172 10.1016/j.jss.2024.04.075 38815515

[15] Fletcher-Sandersjöö A, Tatter C, Tjerkaski J, Bartek J Jr, Maegele M, Nelson DW, Svensson M, Thelin EP, Bellander B-M (2023) Time course and clinical significance of hematoma expansion in moderate-to-severe traumatic brain injury: an observational cohort study. Neurocrit Care 38(1):60–70 10.1007/s12028-022-01609-w PMC9935722 36167951

[16] Forte ML, Brandt S, Claussen AM, Tignanelli CJ, Shah S, Butler M (2025) Venous thromboembolism prophylaxis in adults with acute traumatic brain injury: a systematic review. Trauma Surg Acute Care Open 10(2):e001691 10.1136/tsaco-2024-001691 PMC12184397 40552210

[17] Garcia C, Karamian A, Kotzur T, Gunaji R, Lajeunesse B, Moran M, Martinez P, Chen K, Seifi A (2025) Pulmonary embolism in patients with traumatic brain injury in the United States during 2016–2020. Brain Inj 39(14):1252–1261 10.1080/02699052.2025.2549941 40873010

[18] Geerts WH, Code KI, Jay RM, Chen E, Szalai JP (1994) A prospective study of venous thromboembolism after major trauma. N Engl J Med 331(24):1601–1606 10.1056/NEJM199412153312401 7969340

[19] Hachem LD, Mansouri A, Scales DC, Geerts W, Pirouzmand F (2018) Anticoagulant prophylaxis against venous thromboembolism following severe traumatic brain injury: a prospective observational study and systematic review of the literature. Clin Neurol Neurosurg 175:68–73 10.1016/j.clineuro.2018.09.032 30384119

[20] Hale K, Phar, BCPS, Washington (2024) A review of moderate and severe traumatic brain injury. https://www.uspharmacist.com/article/a-review-of-moderate-and-severe-traumatic-brain-injury. Accessed 5 July 2026

[21] Higgins JPT, Thompson SG, Deeks JJ, Altman DG (2003) Measuring inconsistency in meta-analyses. BMJ 327(7414):557–560 10.1136/bmj.327.7414.557 PMC192859 12958120

[22] Jamjoom AAB, Chari A, Salijevska J, Meacher R, Brennan P, Statham P (2016) A national survey of thromboprophylaxis in traumatic brain injury in the United Kingdom. Br J Neurosurg 30(2):240–245 10.3109/02688697.2016.1161170 26999404

[23] Jamjoom AAB, Jamjoom AB (2013) Safety and efficacy of early pharmacological thromboprophylaxis in traumatic brain injury: systematic review and meta-analysis. J Neurotrauma 30(7):503–511 10.1089/neu.2012.2584 23517138

[24] Karmur BS, Mann JA, Leung AA, Kramer AH, Yang MMH (2026) Early versus late initiation of chemical venous thromboembolism prophylaxis in adult patients with severe traumatic brain injury: a systematic review and meta-analysis. Neurocrit Care 44(2):427–444 10.1007/s12028-025-02404-z 41239163

[25] Kim L, Schuster J, Holena DN, Sims CA, Levine J, Pascual JL (2014) Early initiation of prophylactic heparin in severe traumatic brain injury is associated with accelerated improvement on brain imaging. J Emerg Trauma Shock 7(3):141–148 10.4103/0974-2700.136846 PMC4126111 25114421

[26] Konstantinides SV, Meyer G, Becattini C et al (2020) 2019 ESC guidelines for the diagnosis and management of acute pulmonary embolism developed in collaboration with the European Respiratory Society (ERS). Eur Heart J 41(4):543–603 10.1093/eurheartj/ehz405 31504429

[27] Lu VM, Alvi MA, Rovin RA, Kasper EM (2020) Clinical outcomes following early versus late pharmacologic thromboprophylaxis in patients with traumatic intracranial hemorrhage: a systematic review and meta-analysis. Neurosurg Rev 43(3):861–872 10.1007/s10143-018-1045-y 30374758

[28] Maas AIR, Menon DK, Adelson PD et al (2017) Traumatic brain injury: integrated approaches to improve prevention, clinical care, and research. Lancet Neurol 16(12):987–1048 10.1016/S1474-4422(17)30371-X 29122524

[29] Maegele M, Schöchl H, Menovsky T, Maréchal H, Marklund N, Buki A, Stanworth S (2017) Coagulopathy and haemorrhagic progression in traumatic brain injury: advances in mechanisms, diagnosis, and management. Lancet Neurol 16(8):630–647 10.1016/S1474-4422(17)30197-7 28721927

[30] Margolick J, Dandurand C, Duncan K, Chen W, Evans DC, Sekhon MS, Garraway N, Griesdale DEG, Gooderham P, Hameed SM (2018) A systematic review of the risks and benefits of venous thromboembolism prophylaxis in traumatic brain injury. Can J Neurol Sci 45(4):432–444 10.1017/cjn.2017.275 29895339

[31] Mesa Galan LA, Egea-Guerrero JJ, Quintana Diaz M, Vilches-Arenas A (2016) The effectiveness and safety of pharmacological prophylaxis against venous thromboembolism in patients with moderate to severe traumatic brain injury: a systematic review and meta-analysis. J Trauma Acute Care Surg 81(3):567–574 10.1097/TA.0000000000001134 27244574

[32] Minges KE, Bikdeli B, Wang Y, Attaran RR, Krumholz HM (2018) National and regional trends in deep vein thrombosis hospitalization rates, discharge disposition, and outcomes for medicare beneficiaries. Am J Med 131(10):1200–1208 10.1016/j.amjmed.2018.04.033 PMC7040884 29753792

[33] Monteiro M, Newcombe VFJ, Mathieu F et al (2020) Multiclass semantic segmentation and quantification of traumatic brain injury lesions on head CT using deep learning: an algorithm development and multicentre validation study. Lancet Digit Health 2(6):e314–e322 10.1016/S2589-7500(20)30085-6 33328125

[34] Nyquist P, Bautista C, Jichici D et al (2016) Prophylaxis of venous thrombosis in neurocritical care patients: an evidence-based guideline: a statement for healthcare professionals from the Neurocritical Care Society. Neurocrit Care 24(1):47–60 10.1007/s12028-015-0221-y 26646118

[35] O’Neil ME, Carlson K, Storzbach D, Brenner L, Freeman M, Quiñones A, Motu’apuaka M, Ensley M, Kansagara D (2013) Complications of mild traumatic brain injury in veterans and military personnel: a systematic review 24600749

[36] Ouzzani M, Hammady H, Fedorowicz Z, Elmagarmid A (2016) Rayyan-a web and mobile app for systematic reviews. Syst Rev 5(1):210 10.1186/s13643-016-0384-4 PMC5139140 27919275

[37] Overview | Venous thromboembolism in over 16s: reducing the risk of hospital-acquired deep vein thrombosis or pulmonary embolism | Guidance | NICE

[38] Page MJ, McKenzie JE, Bossuyt PM et al (2021) The PRISMA 2020 statement: an updated guideline for reporting systematic reviews. BMJ 372:n71 10.1136/bmj.n71 PMC8005924 33782057

[39] Pastorek RA, Cripps MW, Bernstein IH, Scott WW, Madden CJ, Rickert KL, Wolf SE, Phelan HA (2014) The Parkland Protocol’s modified Berne-Norwood criteria predict two tiers of risk for traumatic brain injury progression. J Neurotrauma 31(20):1737–1743 10.1089/neu.2014.3366 PMC4180120 24945196

[40] Phelan HA, Wolf SE, Norwood SH et al (2012) A randomized, double-blinded, placebo-controlled pilot trial of anticoagulation in low-risk traumatic brain injury: the Delayed Versus Early Enoxaparin Prophylaxis I (DEEP I) study. J Trauma Acute Care Surg 73(6):1434–1441 10.1097/TA.0b013e31825ac49e 22914079

[41] R Core Team R (2023) R: a language and environment for statistical computing

[42] Scales DC, Riva-Cambrin J, Wells D, Athaide V, Granton JT, Detsky AS (2010) Prophylactic anticoagulation to prevent venous thromboembolism in traumatic intracranial hemorrhage: a decision analysis. Crit Care 14(2):R72 10.1186/cc8980 PMC2887195 20406444

[43] Schünemann HJ, Oxman AD, Brozek J et al (2008) Grading quality of evidence and strength of recommendations for diagnostic tests and strategies. BMJ 336(7653):1106–1110 10.1136/bmj.39500.677199.AE PMC2386626 18483053

[44] Seifi A, Dengler B, Martinez P, Godoy DA (2018) Pulmonary embolism in severe traumatic brain injury. J Clin Neurosci 57:46–50 10.1016/j.jocn.2018.08.042 30154000

[45] Shuja MH, Nasir MM, Mushahid H et al (2025) A systematic review and meta-analysis on the impact of early vs. delayed pharmacological thromboprophylaxis in patients with traumatic brain injury. J Clin Neurosci 132(110936):110936 10.1016/j.jocn.2024.110936 39662114

[46] Skrifvars MB, Bailey M, Presneill J et al (2017) Venous thromboembolic events in critically ill traumatic brain injury patients. Intensive Care Med 43(3):419–428 10.1007/s00134-016-4655-2 28028552

[47] Spano PJ 2nd, Shaikh S, Boneva D, Hai S, McKenney M, Elkbuli A (2020) Anticoagulant chemoprophylaxis in patients with traumatic brain injuries: a systematic review. J Trauma Acute Care Surg 88(3):454–460 10.1097/TA.0000000000002580 31923051

[48] Sterne JA, Hernán MA, Reeves BC et al (2016) ROBINS-I: a tool for assessing risk of bias in non-randomised studies of interventions. BMJ 355:i4919 10.1136/bmj.i4919 PMC5062054 27733354

[49] Stocchetti N, Carbonara M, Citerio G, Ercole A, Skrifvars MB, Smielewski P, Zoerle T, Menon DK (2017) Severe traumatic brain injury: targeted management in the intensive care unit. Lancet Neurol 16(6):452–464 10.1016/S1474-4422(17)30118-7 28504109

[50] Vrettou CS, Dima E, Karela NR, Sigala I, Korfias S (2024) Severe traumatic brain injury and pulmonary embolism: risks, prevention, diagnosis and management. J Clin Med 13(15):4527 10.3390/jcm13154527 PMC11313609 39124793

[51] Zhong H, Feng Y, Shen J, Rao T, Dai H, Zhong W, Zhao G (2025) Global burden of traumatic brain injury in 204 countries and territories from 1990 to 2021. Am J Prev Med 68(4):754–763 10.1016/j.amepre.2025.01.001 39793770

[52] Zhou L, Xue X, Chen Q, Xin Y, Xiao Y (2025) Effect of very early (<24 hours) pharmacological thromboprophylaxis on clinical outcome in patients with traumatic intracranial hemorrhage: a systematic review and meta-analysis. World Neurosurg 202(124419):124419 10.1016/j.wneu.2025.124419 40876746

