# Supplementary material for: Timing of pharmacological thromboprophylaxis in severe traumatic brain injury: a systematic review and meta-analysis
Source: Acta Neurochir (Wien). 2026 Aug 14;168(1):261. doi: 10.1007/s00701-026-07002-2 (PMC13751730; doi:10.1007/s00701-026-07002-2)
Supplement: Supplementary file 1 [file 701_2026_7002_MOESM1_ESM.docx]

# Supplementary Material

| Database | Search Strategy |
| --- | --- |
| MEDLINE (via Ovid), 1946 to 1 May 2026  (n=1076) | 1. exp Brain Injuries, Traumatic/  2. exp Craniocerebral Trauma/  3. (traumatic brain injur* or TBI or head injur* or intracranial hemorrhag* or intracranial haemorrhag*).ti,ab.  4. 1 or 2 or 3    5. exp Venous Thromboembolism/  6. exp Venous Thrombosis/  7. exp Pulmonary Embolism/  8. (venous thromboembol* or VTE or deep vein thromb* or DVT or pulmonary embol* or thromboembol*).ti,ab.  9. 5 or 6 or 7 or 8    10. exp Anticoagulants/  11. exp Heparin/  12. exp Heparin, Low-Molecular-Weight/  13. exp Enoxaparin/  14. (thromboprophylaxis or chemoprophylaxis or anticoagul* or heparin* or LMWH or enoxaparin* or unfractionated heparin or fondaparinux or dalteparin).ti,ab.  15. 10 or 11 or 12 or 13 or 14    16. 4 and 9 and 15  17. limit 16 to (humans and english) |
| EMBASE (via Ovid), 1974 to 1 May 2026  (n=3604) | 1. exp brain injury/  2. exp head injury/  3. (traumatic brain injur* or TBI or head injur* or intracranial hemorrhag* or intracranial haemorrhag*).ti,ab.  4. 1 or 2 or 3    5. exp venous thromboembolism/  6. exp deep vein thrombosis/  7. exp lung embolism/  8. (venous thromboembol* or VTE or deep vein thromb* or DVT or pulmonary embol* or thromboembol*).ti,ab.  9. 5 or 6 or 7 or 8    10. exp anticoagulant agent/  11. exp heparin/  12. exp low molecular weight heparin/  13. exp enoxaparin/  14. (thromboprophylaxis or chemoprophylaxis or anticoagul* or heparin* or LMWH or enoxaparin* or unfractionated heparin or fondaparinux or dalteparin).ti,ab.  15. 10 or 11 or 12 or 13 or 14    16. 4 and 9 and 15  17. limit 16 to (human and english) |
| CENTRAL (Cochrane Library), to 1 May 2026  (n=326) | #1 MeSH descriptor: [Brain Injuries, Traumatic] explode all trees  #2 MeSH descriptor: [Craniocerebral Trauma] explode all trees  #3 (traumatic brain injur* OR TBI OR head injur* OR intracranial hemorrhag* OR intracranial haemorrhag*):ti,ab,kw  #4 #1 OR #2 OR #3    #5 MeSH descriptor: [Venous Thromboembolism] explode all trees  #6 MeSH descriptor: [Venous Thrombosis] explode all trees  #7 MeSH descriptor: [Pulmonary Embolism] explode all trees  #8 (venous thromboembol* OR VTE OR deep vein thromb* OR DVT OR pulmonary embol* OR thromboembol*):ti,ab,kw  #9 #5 OR #6 OR #7 OR #8    #10 MeSH descriptor: [Anticoagulants] explode all trees  #11 MeSH descriptor: [Heparin] explode all trees  #12 MeSH descriptor: [Heparin, Low-Molecular-Weight] explode all trees  #13 MeSH descriptor: [Enoxaparin] explode all trees  #14 (thromboprophylaxis OR chemoprophylaxis OR anticoagul* OR heparin* OR LMWH OR enoxaparin* OR unfractionated heparin OR fondaparinux OR dalteparin):ti,ab,kw  #15 #10 OR #11 OR #12 OR #13 OR #14    #16 #4 AND #9 AND #15  Filters applied at the results stage: English language; restrict result type to "Trials". |
| CDSR (Cochrane Library), to 1 May 2026 | ("traumatic brain injur*" OR "head injur*" OR TBI) AND  ("venous thromboembol*" OR "deep vein thromb*" OR "pulmonary embol*"  OR thromboprophylaxis OR heparin* OR enoxaparin*)  Search fields: title, abstract, keyword (:ti,ab,kw). Result type restricted to "Cochrane Reviews" and "Protocols". |
| PROSPERO, to 1 May 2026 | ("traumatic brain injury" OR "head injury" OR TBI) AND  ("thromboprophylaxis" OR "venous thromboembolism" OR "DVT" OR "heparin" OR "enoxaparin")  Searched in the PROSPERO basic search field (which queries Title, Review question, and Keywords). |
| Backward citation searching  (n=76) | Reference lists of all included studies and eleven prior systematic reviews |
| Forward citation searching  (n=480) | Google Scholar "Cited by" function applied to eleven prior systematic reviews, filtered to 1 May 2026 |

**Supplementary Table 1: Search Strategies**

Full search strategies used across MEDLINE (via Ovid), EMBASE (via Ovid), CENTRAL, the Cochrane Database of Systematic Reviews (CDSR), and PROSPERO, supplemented by backward and forward citation searching.

n denotes the number of records retrieved per source.

|  | **Seifi 2018** | **Dengler 2016** | **Used in this review** |
| --- | --- | --- | --- |
| Source cohort | UTHSCSA neurosurgical ICU, 2011–2013 | UTHSCSA neurosurgical ICU, 2011–2013 | Single cohort (UTHSCSA, 2011–2013) |
| Sample size | n=155 | n=155 (PE outcome on n=119 subset) | n=155 (PTP arm n=122; no-PTP arm n=33) |
| DVT events | Not reported | 9 in PTP arm | 9 DVT events (Dengler 2016) |
| PE events | 4 in PTP arm, all with concurrent DVT | Not reported separately | Not double-counted; composite VTE = 9 |
| ICH progression | Not reported | 12/122 (PTP) vs 0/33 (no-PTP) | Taken from Dengler 2016 |
| Mortality | Reported (identical to Dengler) | Reported (identical to Seifi) | Taken from Dengler 2016 for consistency |

**Supplementary Table 2: Cohort overlap mapping for the Seifi 2018 and Dengler 2016 reports.**

The Seifi 2018 and Dengler 2016 reports describe the same single-centre cohort enrolled at the University of Texas Health Science Center at San Antonio (UTHSCSA) between 2011 and 2013. The table details the per-outcome deduplication rule applied throughout this review, specifying which data were extracted from each report to avoid double-counting of participants.

| **Cohort removed** | **Primary VTE, OR (95% CI)** | **Comment** | **DVT-only, OR (95% CI)** | **Comment** | **Mortality, OR (95% CI)** | **Comment** |
| --- | --- | --- | --- | --- | --- | --- |
| Byrne 2016 | 0.53 (0.10–2.69) | Significance lost | 0.59 (0.13–2.67) | Significance lost | 0.96 (0.36–2.54) | Null preserved |
| Hachem 2018 | 0.47 (0.17–1.31) | Significance lost | 0.50 (0.20–1.24) | Significance lost | 1.14 (0.82–1.60) | Null preserved |
| Elkbuli 2024 | 0.53 (0.33–0.85) | Significance preserved | 0.54 (0.36–0.83) | Significance preserved | 0.94 (0.46–1.90) | Null preserved |
| Kim L 2014 | 0.46 (0.31–0.68) | Significance preserved | 0.49 (0.35–0.68) | Significance preserved | 1.17 (0.94–1.46) | Null preserved |

**Supplementary Table 3: Leave-one-out sensitivity analysis.**

Pooled odds ratios (ORs) for venous thromboembolism (early versus late prophylaxis), deep vein thrombosis (DVT)-only sensitivity analysis, and in-hospital mortality, re-estimated after sequential removal of each of the four contributing cohorts.

**Supplementary Figure 1: Forest plot of deep vein thrombosis incidence comparing early versus late pharmacological thromboprophylaxis in adults with severe traumatic brain injury (DVT-only sensitivity analysis).**

Individual study odds ratios and 95% confidence intervals are shown; the diamond represents the pooled random-effects estimate.

Abbreviations: CI = confidence interval; DVT = deep vein thrombosis; OR = odds ratio.

**Supplementary Figure 2: Forest plot of venous thromboembolism incidence comparing pharmacological thromboprophylaxis plus mechanical thromboprophylaxis versus mechanical thromboprophylaxis alone in adults with severe traumatic brain injury.**

Individual study odds ratios and 95% confidence intervals are shown; the diamond represents the pooled random-effects estimate.

Abbreviations: CI = confidence interval; OR = odds ratio; PTP = pharmacological thromboprophylaxis; VTE = venous thromboembolism.
